# The genome sequence of the Dusky Thorn moth, *Ennomos fuscantarius *(Haworth, 1809)

**DOI:** 10.12688/wellcomeopenres.20174.2

**Published:** 2026-02-14

**Authors:** Douglas Boyes, Dominic Phillips, University of Oxford and Wytham Woods Genome Acquisition Lab, Darwin Tree of Life Barcoding collective, Wellcome Sanger Institute Tree of Life programme, Wellcome Sanger Institute Scientific Operations: DNA Pipelines collective, Tree of Life Core Informatics collective, Darwin Tree of Life Consortium

**Affiliations:** 1 UK Centre for Ecology & Hydrology, Wallingford, England, UK; 2 Natural History Museum, London, England, UK

**Keywords:** Ennomos fuscantarius, Dusky Thorn, genome sequence, chromosomal, Lepidoptera

## Abstract

We present a genome assembly from an individual male *Ennomos fuscantarius* (the Dusky Thorn; Arthropoda; Insecta; Lepidoptera; Geometridae). The genome sequence is 444.9 megabases in span. Most of the assembly is scaffolded into 31 chromosomal pseudomolecules, including the Z sex chromosome. The mitochondrial genome has also been assembled and is 15.49 kilobases in length. Gene annotation of this assembly on Ensembl identified 12,173 protein coding genes.

## Species taxonomy

Eukaryota; Metazoa; Eumetazoa; Bilateria; Protostomia; Ecdysozoa; Panarthropoda; Arthropoda; Mandibulata; Pancrustacea; Hexapoda; Insecta; Dicondylia; Pterygota; Neoptera; Endopterygota; Amphiesmenoptera; Lepidoptera; Glossata; Neolepidoptera; Heteroneura; Ditrysia; Obtectomera; Geometroidea; Geometridae; Ennominae; *Ennomos; Ennomos fuscantaria* (Haworth, 1809) (NCBI:txid722662).

## Background

*Ennomos fuscantaria* (Dusky Thorn) is a Geometrid moth in the Ennominae subfamily with a forewing length of 17–21 mm; a greyish or mauve-shaded band between the forewing margin and distal cross-line distinguishes it from similar species ( Lewis, 2023; Waring *et al.*, 2017).

*E. fuscantaria* is a monovoltine species, flying from late July to early October. Though it readily comes to light, it is not often seen out on the wing ( Randle *et al.*, 2019; Waring *et al.*, 2017). The larva feeds predominantly on ash ( *Fraxinus excelsior*), on which it overwinters as an egg, later to pupate within spun leaves. It can be coaxed to feed on privets ( *Ligustrum* spp.) ( Waring *et al.*, 2017).

*E. fuscantaria* can be found in most habitats where ash trees are present; in the UK, it has a widespread distribution, frequently occurring through England and Wales ( Randle *et al.*, 2019). Globally, *E. fuscantaria* is distributed throughout the western Palearctic, extending from western Europe to eastern Russia and the Mediterranean ( GBIF Secretariat, 2023).

Numbers of *E. fuscantaria* are prone to fluctuate in the UK and are currently present on the Rothamstead Red List, with suspected population numbers decreasing by of 47% over a 10-year period as of 2019, with earlier research showing a 90% decrease between 1990 and 2001 ( GBIF Secretariat, 2023; Palmer *et al.*, 2015; Waring *et al.*, 2017). This species is highly sensitive to the progression of climate change, as demonstrate by the large decrease in population linked to increased rates of ash dieback ( Fox *et al.*, 2021; Palmer *et al.*, 2015). The genome sequence of *E. fuscantaria* will provide insights into how we can help to reduce these impacts that changes in climate can cause to occupancy and distribution of this vulnerable species. Sequence data will also support phylogenetic analyses of the relationships between Ennominae moths and studies of the evolution of flightlessness within the genus ( Wahlberg *et al.*, 2010).

The genome of *Ennomos fuscantarius* was sequenced as part of the Darwin Tree of Life Project, a collaborative effort to sequence all named eukaryotic species in the Atlantic Archipelago of Britain and Ireland. Here we present a chromosomally complete genome sequence for *Ennomos fuscantarius*, based on one specimen collected by Douglas Boyes from Wytham Woods, Oxfordshire.

## Genome sequence report

**Figure 1.**
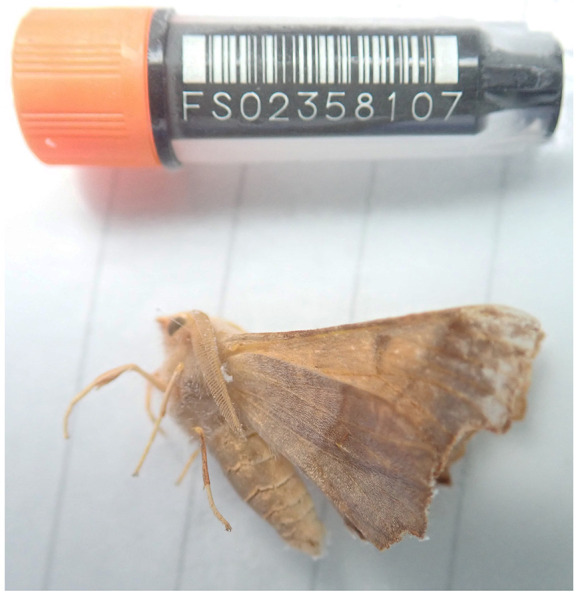
Photograph of the *Ennomos fuscantarius* (ilEnnFusc2) specimen used for genome sequencing.

The genome was sequenced from one male *Ennomos fuscantarius* ( Figure 1) collected from Wytham Woods, UK (51.77, –1.34). A total of 52-fold coverage in Pacific Biosciences single-molecule HiFi long reads and 85-fold coverage in 10X Genomics read clouds were generated. Primary assembly contigs were scaffolded with chromosome conformation Hi-C data. Manual assembly curation corrected 12 missing joins or mis-joins, reducing the scaffold number by 13.95%, and increasing the scaffold N50 by 1.61%.

**Figure 2.**
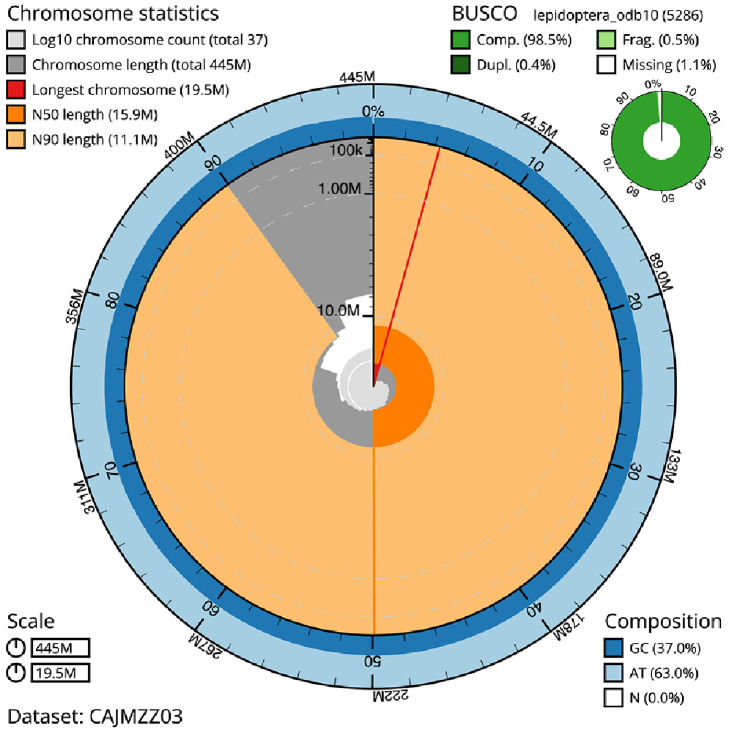
Genome assembly of *Ennomos fuscantarius*, ilEnnFusc2.3: metrics. The BlobToolKit Snailplot shows N50 metrics and BUSCO gene completeness. The main plot is divided into 1,000 size-ordered bins around the circumference with each bin representing 0.1% of the 444,925,525 bp assembly. The distribution of scaffold lengths is shown in dark grey with the plot radius scaled to the longest scaffold present in the assembly (19,450,406 bp, shown in red). Orange and pale-orange arcs show the N50 and N90 scaffold lengths (15,884,173 and 11,089,215 bp), respectively. The pale grey spiral shows the cumulative scaffold count on a log scale with white scale lines showing successive orders of magnitude. The blue and pale-blue area around the outside of the plot shows the distribution of GC, AT and N percentages in the same bins as the inner plot. A summary of complete, fragmented, duplicated and missing BUSCO genes in the lepidoptera_odb10 set is shown in the top right. An interactive version of this figure is available at https://blobtoolkit.genomehubs.org/view/ilEnnFusc2.3/dataset/CAJMZZ03/snail.

**Figure 3.**
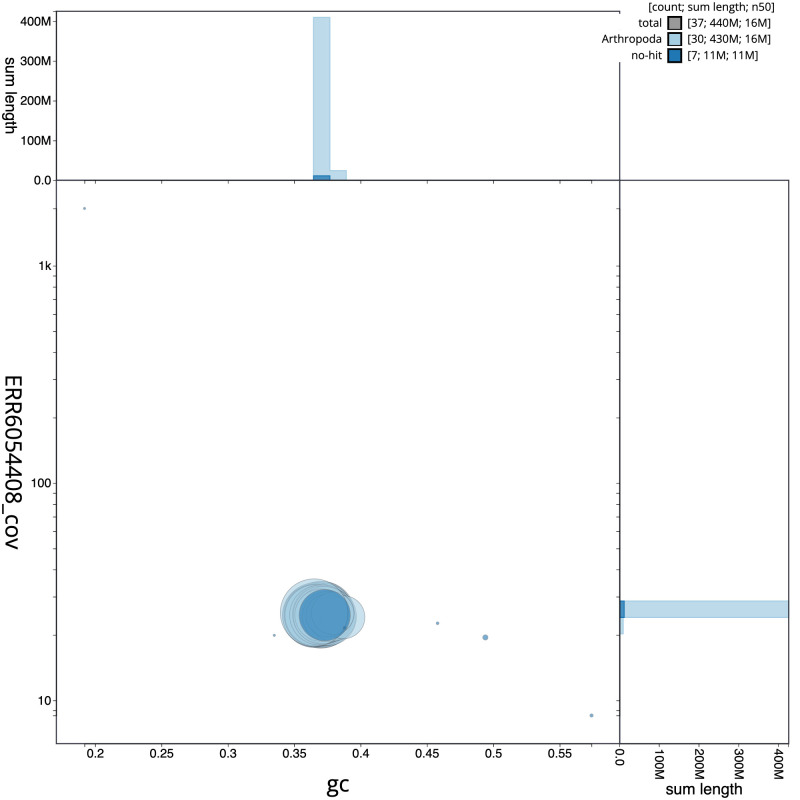
Genome assembly of *Ennomos fuscantarius*, ilEnnFusc2.3: BlobToolKit GC-coverage plot. Scaffolds are coloured by phylum. Circles are sized in proportion to scaffold length. Histograms show the distribution of scaffold length sum along each axis. An interactive version of this figure is available at https://blobtoolkit.genomehubs.org/view/ilEnnFusc2.3/dataset/CAJMZZ03/blob.

**Figure 4.**
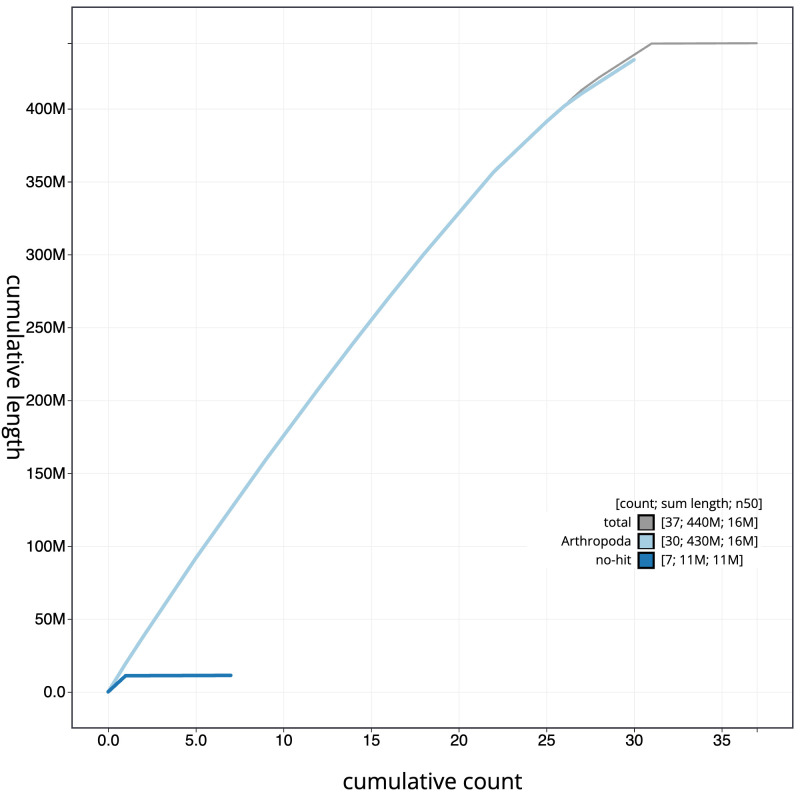
Genome assembly of *Ennomos fuscantarius*, ilEnnFusc2.3: BlobToolKit cumulative sequence plot. The grey line shows cumulative length for all scaffolds. Coloured lines show cumulative lengths of scaffolds assigned to each phylum using the buscogenes taxrule. An interactive version of this figure is available at https://blobtoolkit.genomehubs.org/view/ilEnnFusc2.3/dataset/CAJMZZ03/cumulative.

**Figure 5.**
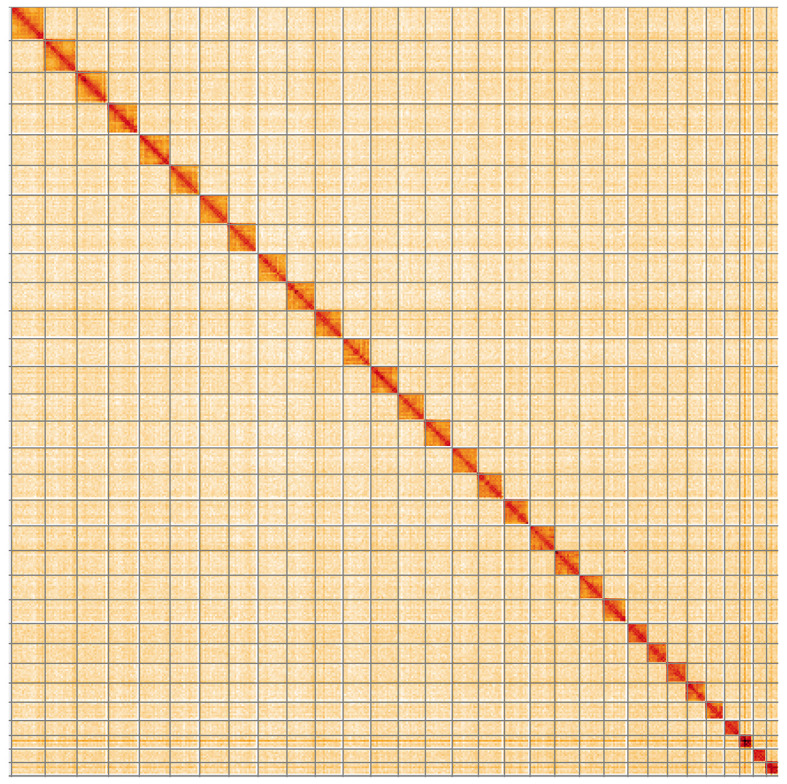
Genome assembly of *Ennomos fuscantarius*, ilEnnFusc2.3: Hi-C contact map of the ilEnnFusc2.3 assembly, visualised using HiGlass. Chromosomes are shown in order of size from left to right and top to bottom. An interactive version of this figure may be viewed at https://genome-note-higlass.tol.sanger.ac.uk/l/?d=KGN1bRa6S8ahRJaRSp9yrA.

The final assembly has a total length of 444.9 Mb in 36 sequence scaffolds with a scaffold N50 of 15.9 Mb ( Table 1). A summary of the assembly statistics is shown in Figure 2, while the distribution of assembly scaffolds on GC proportion and coverage is shown in Figure 3. The cumulative assembly plot in Figure 4 shows curves for subsets of scaffolds assigned to different phyla. Most (99.95%) of the assembly sequence was assigned to 31 chromosomal-level scaffolds, representing 30 autosomes and the Z sex chromosome. Chromosome-scale scaffolds confirmed by the Hi-C data are named in order of size ( Figure 5; Table 2). While not fully phased, the assembly deposited is of one haplotype. Contigs corresponding to the second haplotype have also been deposited. The mitochondrial genome was also assembled and can be found as a contig within the multifasta file of the genome submission.

**Table 1.** Genome data for *Ennomos fuscantarius*, ilEnnFusc2.3.

Project accession data		
Assembly identifier	ilEnnFusc2.3	
Species	*Ennomos fuscantarius*	
Specimen	ilEnnFusc2	
NCBI taxonomy ID	722662	
BioProject	PRJEB42951	
BioSample ID	SAMEA7520185	
Isolate information	ilEnnFusc2: male: head and thorax (PacBio HiFi), abdomen (Hi-C scaffolding) ilEnnFusc1: whole organism (RNA sequencing)	
Assembly metrics *		***Benchmark***
Consensus quality (QV)	Primary: 56.3; alternate: 54.4; combined: 55.0	≥ *40*
*k*-mer completeness	Primary: 96.74%; alternate: 81.22%; combined: 99.33%	≥ *95%*
BUSCO **	C:98.5%[S:98.1%,D:0.4%],F:0.5%,M:1.1%,n:5,286	*C* ≥ *95%*
Percentage of assembly mapped to chromosomes	99.95%	≥ *90%*
Sex chromosomes	Z chromosome	*localised homologous pairs*
Organelles	Mitochondrial genome assembled	*complete single alleles*
**Raw data accessions**		
PacificBiosciences SEQUEL II	ERR6560797	
10X Genomics Illumina	ERR6054405, ERR6054407, ERR6054408, ERR6054406	
Hi-C Illumina	ERR6054409	
PolyA RNA-Seq Illumina	ERR6054410, ERR6787420	
**Genome assembly**		
Assembly accession	GCA_905220475.3	
*Accession of alternate haplotype*	GCA_905220485.1	
Span (Mb)	444.9	
Number of contigs	46	
Contig N50 length (Mb)	15.5	
Number of scaffolds	36	
Scaffold N50 length (Mb)	15.9	
Longest scaffold (Mb)	19.5	
**Genome annotation**		
Number of protein-coding genes	12,173	
Number of non-coding genes	1,740	
Number of gene transcripts	23,475	

* Assembly metric benchmarks are adapted from column VGP-2020 of “Table 1: Proposed standards and metrics for defining genome assembly quality” from ( Rhie *et al.*, 2021).

** BUSCO scores based on the lepidoptera_odb10 BUSCO set using v5.3.2. C = complete [S = single copy, D = duplicated], F = fragmented, M = missing, n = number of orthologues in comparison. A full set of BUSCO scores is available at https://blobtoolkit.genomehubs.org/view/ilEnnFusc2.3/dataset/CAJMZZ03/busco.

**Table 2.** Chromosomal pseudomolecules in the genome assembly of *Ennomos fuscantarius*, ilEnnFusc2.

INSDC accession	Chromosome	Length (Mb)	GC%
HG992024.1	1	18.42	37.0
HG992025.1	2	18.21	37.0
HG992026.1	3	17.84	37.0
HG992027.1	4	17.76	37.0
HG992028.1	5	17.17	36.5
HG992029.1	6	16.98	36.5
HG992030.1	7	16.84	36.5
HG992031.1	8	16.68	36.5
HG992032.1	9	16.42	36.5
HG992033.1	10	16.09	37.0
HG992034.1	11	16.07	36.5
HG992035.1	12	15.88	37.0
HG992036.1	13	15.7	36.5
HG992037.1	14	15.53	37.0
HG992038.1	15	15.19	37.0
HG992039.1	16	15.06	37.0
HG992040.1	17	14.83	37.0
HG992042.1	19	14.29	37.0
HG992041.1	18	14.29	37.5
HG992043.1	20	14.18	37.0
HG992044.1	21	13.83	37.5
HG992045.1	22	11.57	37.5
HG992046.1	23	11.43	37.0
HG992047.1	24	11.38	37.0
HG992048.1	25	11.09	37.5
HG992049.1	26	10.57	37.5
HG992050.1	27	8.69	38.0
HG992051.1	28	7.82	38.5
HG992052.1	29	7.77	37.5
HG992053.1	30	7.68	38.0
HG992023.1	Z	19.45	36.5
HG992054.3	MT	0.02	19.5

The combined primary and alternate assemblies achieve an estimated QV of 55.0. The *k*-mer completeness is 96.74% for the primary assembly, 81.22% for the alternate haplotype, and 99.33% for the combined assemblies. The primary assembly has a BUSCO v5.3.2 completeness of 98.5% (single = 98.1%, duplicated = 0.4%), using the lepidoptera_odb10 reference set ( *n* = 5,286).

Metadata for specimens, spectral estimates, sequencing runs, contaminants and pre-curation assembly statistics can be found at https://links.tol.sanger.ac.uk/species/722662.

## Genome annotation report

The *Ennomos fuscantarius* genome assembly (GCA_905220475.3) was annotated using the Ensembl rapid annotation pipeline. The resulting annotation includes 23,475 transcribed mRNAs from 12,173 protein-coding and 1,740 non-coding genes. Further details of this annotation are available from the Ensembl annotation page.

## Methods

### Sample acquisition and nucleic acid extraction

A male *Ennomos fuscantarius* (specimen ID Ox000200, individual ilEnnFusc2) was collected from Wytham Woods, Oxfordshire (biological vice-county Berkshire), UK (latitude 51.77, longitude –1.34) on 2019-08-24 using a light trap. The specimen was collected and identified by Douglas Boyes (University of Oxford) and preserved on dry ice.

DNA was extracted at the Tree of Life laboratory, Wellcome Sanger Institute (WSI). The ilEnnFusc2 sample was weighed and dissected on dry ice with tissue set aside for Hi-C sequencing. Head and thorax tissue was disrupted using a Nippi Powermasher fitted with a BioMasher pestle. High molecular weight (HMW) DNA was extracted using the Qiagen MagAttract HMW DNA extraction kit. Low molecular weight DNA was removed from a 20 ng aliquot of extracted DNA using the 0.8X AMpure XP purification kit prior to 10X Chromium sequencing; a minimum of 50 ng DNA was submitted for 10X sequencing. HMW DNA was sheared into an average fragment size of 12–20 kb in a Megaruptor 3 system with speed setting 30. Sheared DNA was purified by solid-phase reversible immobilisation using AMPure PB beads with a 1.8X ratio of beads to sample to remove the shorter fragments and concentrate the DNA sample. The concentration of the sheared and purified DNA was assessed using a Nanodrop spectrophotometer and Qubit Fluorometer and Qubit dsDNA High Sensitivity Assay kit. Fragment size distribution was evaluated by running the sample on the FemtoPulse system.

RNA was extracted from whole organism tissue of ilEnnFusc1 in the Tree of Life Laboratory at the WSI using TRIzol, according to the manufacturer’s instructions. RNA was then eluted in 50 μl RNAse-free water and its concentration assessed using a Nanodrop spectrophotometer and Qubit Fluorometer using the Qubit RNA Broad-Range (BR) Assay kit. Analysis of the integrity of the RNA was done using Agilent RNA 6000 Pico Kit and Eukaryotic Total RNA assay.

### Sequencing

Pacific Biosciences HiFi circular consensus and 10X Genomics read cloud DNA sequencing libraries were constructed according to the manufacturers’ instructions. Poly(A) RNA-Seq libraries were constructed using the NEB Ultra II RNA Library Prep kit. DNA and RNA sequencing was performed by the Scientific Operations core at the WSI on Pacific Biosciences SEQUEL II (HiFi), Illumina HiSeq 4000, Illumina HiSeq 4000 (RNA-Seq) and HiSeq X Ten (10X) instruments. Hi-C data were also generated from abdomen tissue of ilEnnFusc2 using the Arima2 kit and sequenced on the HiSeq X Ten instrument.

### Genome assembly, curation and evaluation

Assembly was carried out with Hifiasm ( Cheng *et al.*, 2021) and haplotypic duplication was identified and removed with purge_dups ( Guan *et al.*, 2020). One round of polishing was performed by aligning 10X Genomics read data to the assembly with Long Ranger ALIGN, calling variants with FreeBayes ( Garrison & Marth, 2012). The assembly was then scaffolded with Hi-C data ( Rao *et al.*, 2014) using SALSA2 ( Ghurye *et al.*, 2019). The assembly was checked for contamination and corrected using the gEVAL system ( Chow *et al.*, 2016) as described previously ( Howe *et al.*, 2021). Manual curation was performed using gEVAL, HiGlass ( Kerpedjiev *et al.*, 2018) and Pretext ( Harry, 2022). The mitochondrial genome was assembled using MitoHiFi ( Uliano-Silva *et al.*, 2023), which runs MitoFinder ( Allio *et al.*, 2020) or MITOS ( Bernt *et al.*, 2013) and uses these annotations to select the final mitochondrial contig and to ensure the general quality of the sequence.

A Hi-C map for the final assembly was produced using bwa-mem2 ( Vasimuddin *et al.*, 2019) in the Cooler file format (Abdennur & Mirny, 2020). To assess the assembly metrics, the *k*-mer completeness and QV consensus quality values were calculated in Merqury.FK ( Rhie *et al.*, 2020). The genome was analysed within the BlobToolKit environment ( Challis *et al.*, 2020) and BUSCO scores ( Manni *et al.*, 2021; Simão *et al.*, 2015) were calculated.

Table 3 contains a list of relevant software tool versions and sources.

**Table 3.** Software tools: versions and sources.

Software tool	Version	Source
BlobToolKit	4.1.7	https://github.com/blobtoolkit/blobtoolkit
BUSCO	5.3.2	https://gitlab.com/ezlab/busco
FreeBayes	1.3.1-17-gaa2ace8	https://github.com/freebayes/freebayes
gEVAL	N/A	https://geval.org.uk/
Hifiasm	0.12	https://github.com/chhylp123/hifiasm
HiGlass	1.11.6	https://github.com/higlass/higlass
Long Ranger ALIGN	2.2.2	https://support.10xgenomics.com/genome-exome/software/pipelines/latest/advanced/other-pipelines
Merqury	1.1.2	https://github.com/thegenemyers/MERQURY.FK
MitoHiFi	1	https://github.com/marcelauliano/MitoHiFi
PretextView	0.2	https://github.com/wtsi-hpag/PretextView
purge_dups	1.2.3	https://github.com/dfguan/purge_dups
SALSA	2.2	https://github.com/salsa-rs/salsa
sanger-tol/genomenote	v1.0	https://github.com/sanger-tol/genomenote
sanger-tol/readmapping	1.1.0	https://github.com/sanger-tol/readmapping/tree/1.1.0

### Genome annotation

The Ensembl gene annotation system ( Aken *et al.*, 2016) was used to generate annotation for the *Ennomos fuscantarius* assembly (GCA_905220475.3). Annotation was created primarily through alignment of transcriptomic data to the genome, with gap filling via protein-to-genome alignments of a select set of proteins from UniProt ( UniProt Consortium, 2019).

### Wellcome Sanger Institute – Legal and Governance

The materials that have contributed to this genome note have been supplied by a Darwin Tree of Life Partner. The submission of materials by a Darwin Tree of Life Partner is subject to the **‘Darwin Tree of Life Project Sampling Code of Practice’,** which can be found in full on the Darwin Tree of Life website here. By agreeing with and signing up to the Sampling Code of Practice, the Darwin Tree of Life Partner agrees they will meet the legal and ethical requirements and standards set out within this document in respect of all samples acquired for, and supplied to, the Darwin Tree of Life Project.

Further, the Wellcome Sanger Institute employs a process whereby due diligence is carried out proportionate to the nature of the materials themselves, and the circumstances under which they have been/are to be collected and provided for use. The purpose of this is to address and mitigate any potential legal and/or ethical implications of receipt and use of the materials as part of the research project, and to ensure that in doing so we align with best practice wherever possible. The overarching areas of consideration are:

• Ethical review of provenance and sourcing of the material
• Legality of collection, transfer and use (national and international)

Each transfer of samples is further undertaken according to a Research Collaboration Agreement or Material Transfer Agreement entered into by the Darwin Tree of Life Partner, Genome Research Limited (operating as the Wellcome Sanger Institute), and in some circumstances other Darwin Tree of Life collaborators.

## Data Availability

European Nucleotide Archive: *Ennomos fuscantarius* (dusky thorn). Accession number PRJEB42951; https://identifiers.org/ena.embl/PRJEB42951. ( Wellcome Sanger Institute, 2021) The genome sequence is released openly for reuse. The *Ennomos fuscantarius* genome sequencing initiative is part of the Darwin Tree of Life (DToL) project. All raw sequence data and the assembly have been deposited in INSDC databases. Raw data and assembly accession identifiers are reported in Table 1. Members of the University of Oxford and Wytham Woods Genome Acquisition Lab are listed here: https://doi.org/10.5281/zenodo.4789928. Members of the Darwin Tree of Life Barcoding collective are listed here: https://doi.org/10.5281/zenodo.4893703. Members of the Wellcome Sanger Institute Tree of Life programme are listed here: https://doi.org/10.5281/zenodo.4783585. Members of Wellcome Sanger Institute Scientific Operations: DNA Pipelines collective are listed here: https://doi.org/10.5281/zenodo.4790455. Members of the Tree of Life Core Informatics collective are listed here: https://doi.org/10.5281/zenodo.5013541. Members of the Darwin Tree of Life Consortium are listed here: https://doi.org/10.5281/zenodo.4783558.
