# Supplementary material for: The genome sequence of the Dusky Thorn moth, Ennomos fuscantarius (Haworth, 1809)
Source: Wellcome Open Res. 2026 Feb 14;8:505. Originally published 2023 Nov 7. [Version 2] doi: 10.12688/wellcomeopenres.20174.2 (PMC12946694; doi:10.12688/wellcomeopenres.20174.2)
Supplement: Peer review history

# Reviewer report: Reviewer response for version 2

**DOI:** 10.21956/wellcomeopenres.27653.r148735

**Published:** 2026-02-26

**Authors:** Stephan Koblmüller

**Affiliations:** 1 University of Graz, Graz, Graz, Austria

In this manuscript, Boyes et al. report the whole-genome assembly of the dusky thorn moth, *Ennomos fuscantarius*, a geometrid species broadly distributed throughout Europe. The authors provide useful background information on the species, which I found quite valuable. The justification for sequencing this species is clearly explained. The data are presented in a clear and organized manner, and the methodological approaches are described in sufficient detail. I have no concerns regarding the validity of the data or the results. As far as I can tell, the associated datasets have been made available in publicly accessible repositories.

Are sufficient details of methods and materials provided to allow replication by others?

Yes

Is the rationale for creating the dataset(s) clearly described?

Yes

Are the datasets clearly presented in a useable and accessible format?

Yes

Are the protocols appropriate and is the work technically sound?

Yes

Reviewer Expertise:

phylegenetics/-genomics, population genetics, phylogeography

I confirm that I have read this submission and believe that I have an appropriate level of expertise to confirm that it is of an acceptable scientific standard.

# Reviewer report: Reviewer response for version 1

**DOI:** 10.21956/wellcomeopenres.22337.r78060

**Published:** 2024-04-30

**Authors:** Saskia Wutke

**Affiliations:** 1 University or Eastern Finland, Joensuu, Finland

This data note reports the genome of the Dusky Thorn (Ennomos fuscantaria), a geometrid moth. The genome was assembled based on PacBio HiFi and 10X Genomics data, scaffolded with Hi-C data, and annotated using transcriptome data. Overall, the dataset is presented clearly and concisely. The methods are state-of-the-art and follow the usual DToL procedure. The final chromosome-level genome assembly is 444.9 Mb long. With a scaffold N50 of 15.9 Mb and a total number of 36 scaffolds, the assembly is of good quality and provides a valuable addition to the growing list of lepidopteran genomes.

 Yet, I have several points that should be addressed.

 TITLE:

 - It is not clear from the title that this is the genome of a moth or even insect. Some kind of information would be helpful.

 BACKGROUND (Paragraph 4):

 - ‘annual variation in climate change’ makes no sense. Please rephrase.

 - Should be ‘as demonstrated’

 - ‘population decrease in population [..]’ 1x population is redundant

 - Please explain how the ash dieback and climate change are connected, as they are mentioned in the same context.

 - ‘full genome’ can only be ‘genome’, full is redundant.

 - ‘these vulnerable species’ should be ‘this vulnerable species’.

 - ‘insights into relationships between Ennominae moths’.

 TABLE 1:

 - Isolate information: ilEnnFusc2: male: head and thorax (should probably be ‘DNA sequencing’ instead of ‘RNA sequencing’)

 METHODS:

 - Sequencing section: Illumina HiSeq 4000 twice (redundant or mistake?)

 - Please provide more details on the transcriptome data used for annotation and the protein set from UniProt. The sex of the individual used for RNA sequencing would be helpful. 

 DATA AVAILABILITY:

 - Details for accessing the genome annotation results/predicted protein set would be helpful.

Are sufficient details of methods and materials provided to allow replication by others?

Partly

Is the rationale for creating the dataset(s) clearly described?

Yes

Are the datasets clearly presented in a useable and accessible format?

Yes

Are the protocols appropriate and is the work technically sound?

Yes

Reviewer Expertise:

Evolutionary genomics, phylogenomics, hymenoptera

I confirm that I have read this submission and believe that I have an appropriate level of expertise to confirm that it is of an acceptable scientific standard, however I have significant reservations, as outlined above.

# Response

**Published:** 2026-02-03

**Authors:** Tree of Life Team Sanger

**Affiliations:** Wellcome Trust Sanger Institute, UK

Thank you for reviewing this data note. 

 We have made the corrections you requested to the text of the Background and to Table 1, and thank you for pointing out these errors. The word "moth" has now been added to the title. We have updated the link to the third-party genome annotation provider: https://beta.ensembl.org/species/aa89cd65-c4eb-4931-aa7d-039604cb00cd

 Tree of Life team

# Reviewer report: Reviewer response for version 1

**DOI:** 10.21956/wellcomeopenres.22337.r71950

**Published:** 2024-01-25

**Authors:** Arjen Van 't Hof

**Affiliations:** 1 Biology Centre of the Czech Academy of Sciences, Budějovice, Czech Republic

The genome assembly of the Dusky Thorn is a valuable contribution to the scientific community, especially since hardly any sequence resources existed for this species. The assembly meets the same high standards as we are used to from the Darwin Tree of Life Consortium with full-length scaffolds for all 31 chromosomes plus a separate assembly for the alternate haplotype. The chromosome number has not been established by karyotyping before to my knowledge. It is consistent with the ancestral Lepidoptera composition of 30 autosomes and one Z chromosome. A male was used for the assembly, which means that the W chromosome is not included. The chromosomes are numbered based on size instead of gene content. This is somewhat surprising because numbering rules which take shared synteny with ancestral chromosomes into account have been proposed.

 The number of identified protein coding genes is lower than reported in other Lepidoptera assemblies, though the BUSCO scores suggest near complete representation.

 RNA was extracted from ilEnnFusc1, a different individual than the male used to obtain DNA. Unfortunately, the sex of ilEnnFusc1 is not specified here or at the sample sheet for SAMEA7520184. The authors mention that the genome assembly can be used to study female lightlessness', and therefore, it would have been useful to know the sex of ilEnnFusc1.

 The Dusky Thorn genome assembly is a welcome addition to a growing number of high-quality Lepidoptera genome sequences.

Are sufficient details of methods and materials provided to allow replication by others?

Yes

Is the rationale for creating the dataset(s) clearly described?

Yes

Are the datasets clearly presented in a useable and accessible format?

Yes

Are the protocols appropriate and is the work technically sound?

Yes

Reviewer Expertise:

Evolutionary Biology

I confirm that I have read this submission and believe that I have an appropriate level of expertise to confirm that it is of an acceptable scientific standard.
